# 1-(3-Chloro­benzo­yl)-3-(2,3-dimethyl­phen­yl)thio­urea

**DOI:** 10.1107/S1600536809000063

**Published:** 2009-01-08

**Authors:** M. Khawar Rauf, Michael Bolte, Amin Badshah

**Affiliations:** a Department of Chemistry, Quaid-i-Azam University, Islamabad 45320, Pakistan; b Institut für Anorganische Chemie, J. W. Goethe-Universität Frankfurt, Max-von-Laue-Strasse 7, 60438 Frankfurt/Main, Germany

## Abstract

The title mol­ecule, C_16_H_15_ClN_2_OS, exists in the solid state in its thione form with typical thio­urea C—S and C—O bonds lengths, as well as shortened C—N bonds. An intra­molecular N—H⋯O hydrogen bond stabilizes the mol­ecular conformation and inter­molecular N—H⋯S hydrogen bonds link the mol­ecules into centrosymmetric dimers. The dihedral angle  between the aromatic rings is 50.18 (5)°.

## Related literature

For related compounds, see: Khawar Rauf *et al.* (2006*a* ▶,*b* ▶,*c* ▶,*d* ▶). For reference bond-length data, see: Allen (2002 ▶).
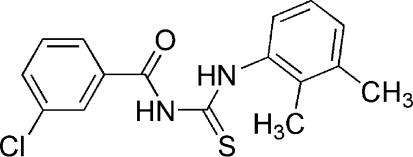


## Experimental

### 

#### Crystal data

C_16_H_15_ClN_2_OS
*M* *_r_* = 318.81
Triclinic, 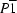

*a* = 8.1315 (9) Å
*b* = 9.3906 (12) Å
*c* = 10.5310 (12) Å
α = 93.296 (8)°
β = 92.623 (8)°
γ = 102.579 (9)°
*V* = 782.14 (16) Å^3^
*Z* = 2
Mo *K*α radiation
μ = 0.38 mm^−1^
*T* = 173 (2) K
0.46 × 0.42 × 0.41 mm

#### Data collection

Stoe IPDS II two-circle diffractometer
Absorption correction: multi-scan (*MULABS*; Spek, 2003 ▶; Blessing, 1995 ▶) *T*_min_ = 0.846, *T*_max_ = 0.861
8257 measured reflections
3066 independent reflections
2846 reflections with *I* > 2σ(*I*)
*R*_int_ = 0.037

#### Refinement

*R*[*F* ^2^ > 2σ(*F* ^2^)] = 0.032
*wR*(*F* ^2^) = 0.085
*S* = 1.04
3066 reflections
201 parameters
H atoms treated by a mixture of independent and constrained refinement
Δρ_max_ = 0.27 e Å^−3^
Δρ_min_ = −0.33 e Å^−3^

### 

Data collection: *X-AREA* (Stoe & Cie, 2001 ▶); cell refinement: *X-AREA*; data reduction: *X-AREA*; program(s) used to solve structure: *SHELXS97* (Sheldrick, 2008 ▶); program(s) used to refine structure: *SHELXL97* (Sheldrick, 2008 ▶); molecular graphics: *XP* in *SHELXTL-Plus* (Sheldrick, 2008 ▶); software used to prepare material for publication: *SHELXL97*.

## Supplementary Material

Crystal structure: contains datablocks I, global. DOI: 10.1107/S1600536809000063/bv2114sup1.cif

Structure factors: contains datablocks I. DOI: 10.1107/S1600536809000063/bv2114Isup2.hkl

Additional supplementary materials:  crystallographic information; 3D view; checkCIF report

## Figures and Tables

**Table 1:** Hydrogen-bond geometry (Å, °)

*D*—H⋯*A*	*D*—H	H⋯*A*	*D*⋯*A*	*D*—H⋯*A*
N2—H2⋯O1	0.86 (2)	1.99 (2)	2.6840 (16)	137.7 (17)
N1—H1⋯S1^i^	0.836 (19)	2.636 (19)	3.4376 (13)	161.2 (15)

Symmetry code: (i) 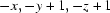.

**Fig. 1.**
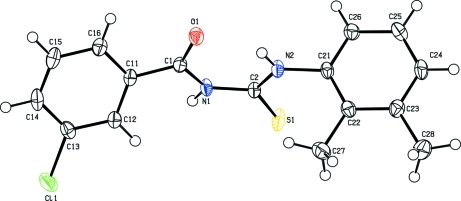
Molecular structure of (I) showing atom numbering scheme. Displacement ellipsoids are drawn at the 50% probability level.

**Fig. 2.**
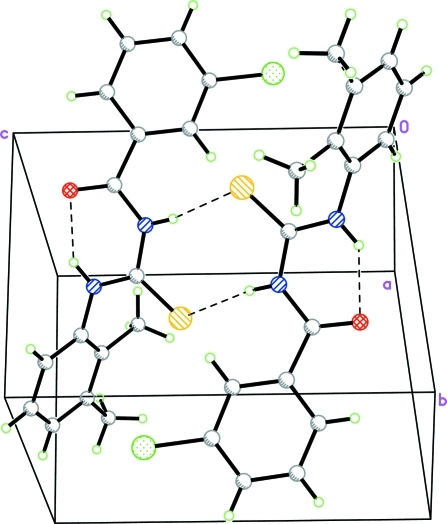
Packing diagram. Hydrogen bonds are shown as dashed lines.

## supplementary crystallographic information

### Comment

The background to this study has been set out in our previous work on the structural chemistry of *N*,*N'*-disubstituted thioureas (Khawar Rauf *et al.*, 2006*a*,*b*,*c*,*d*). Herein, as a continuation of these studies, the structure of the title compound (I) is described. A depiction of the molecule is given in Fig. 1. Bond lengths and angles, see the table of selected geometric parameters, can be regarded as typical for *N*,*N'*-disubstituted thiourea compounds as found in the Cambridge Structural Database v5.28 (Allen, 2002; Khawar Rauf *et al.*, 2006*a*).The molecule exists in the thione form with typical thiourea C—S and C—O bonds, as well as shortened C—N bond lengths. The thiocarbonyl and carbonyl groups are almost coplanar. The dihedral angle between the aromatic rings is 50.18 (5)°. An intramolecular N—H···O hydrogen bond is present (Table 2), forming a six-membered ring commonly observed in this class of compounds (Khawar Rauf *et al.*, 2006*d*). Intermolecular N—H···S hydrogen bonds link the molecules to form centrosymmetric dimers.

### Experimental

Freshly prepared 3-chlorobenzoyl isothiocyanate (2.0 g, 10 mmol) was stirred in acetone (40 ml) for 20 min. Neat 2,3-dimethylaniline (1.62 g, 10 mmol) was then added and the resulting mixture was stirred for 1.5 h. The reaction mixture was then poured into acidified (pH 4) water and stirred well. The solid product was separated and washed with deionized water and purified by recrystallization from methanol–1,1-dichloromethane (1:10 *v/v*) to give fine crystals of (I), with an overall yield of 90%. Full spectroscopic and physical characterization will be reported elsewhere.

### Refinement

H atoms bonded to C were included in calculated positions and refined as riding on their parent C atom with C—H = 0.95 Å *U*_iso_(H) = 1.2*U*_eq_(C) or C—H = 0.98 Å and *U*_iso_(H) = 1.5*U*_eq_(C), respectively, for aromatic and methyl C atoms. The H atoms bonded to N were freely refined.

### Crystal data

**Table:**

C_16_H_15_ClN_2_OS	*Z* = 2
*M**_r_* = 318.81	*F*(000) = 332
Triclinic, *P*1	*D*_x_ = 1.354 Mg m^−^^3^
Hall symbol: -P 1	Mo *K*α radiation, λ = 0.71073 Å
*a* = 8.1315 (9) Å	Cell parameters from 7706 reflections
*b* = 9.3906 (12) Å	θ = 3.7–26.1°
*c* = 10.5310 (12) Å	µ = 0.38 mm^−^^1^
α = 93.296 (8)°	*T* = 173 K
β = 92.623 (8)°	Block, colourless
γ = 102.579 (9)°	0.46 × 0.42 × 0.41 mm
*V* = 782.14 (16) Å^3^	

### Data collection

**Table:**

Stoe IPDS II two-circle diffractometer	3066 independent reflections
Radiation source: fine-focus sealed tube	2846 reflections with *I* > 2σ(*I*)
graphite	*R*_int_ = 0.037
ω scans	θ_max_ = 26.1°, θ_min_ = 3.6°
Absorption correction: multi-scan (*MULABS*; Spek, 2003; Blessing, 1995)	*h* = −10→10
*T*_min_ = 0.846, *T*_max_ = 0.861	*k* = −10→11
8257 measured reflections	*l* = −12→12

### Refinement

**Table:**

Refinement on *F*^2^	Secondary atom site location: difference Fourier map
Least-squares matrix: full	Hydrogen site location: inferred from neighbouring sites
*R*[*F*^2^ > 2σ(*F*^2^)] = 0.032	H atoms treated by a mixture of independent and constrained refinement
*wR*(*F*^2^) = 0.085	*w* = 1/[σ^2^(*F*_o_^2^) + (0.041*P*)^2^ + 0.3782*P*] where *P* = (*F*_o_^2^ + 2*F*_c_^2^)/3
*S* = 1.04	(Δ/σ)_max_ < 0.001
3066 reflections	Δρ_max_ = 0.27 e Å^−^^3^
201 parameters	Δρ_min_ = −0.32 e Å^−^^3^
0 restraints	Extinction correction: *SHELXL97* (Sheldrick, 2008), Fc^*^=kFc[1+0.001xFc^2^λ^3^/sin(2θ)]^-1/4^
Primary atom site location: structure-invariant direct methods	Extinction coefficient: 0.097 (5)

### Special details

**Table:**

Geometry. All e.s.d.'s (except the e.s.d. in the dihedral angle between two l.s. planes) are estimated using the full covariance matrix. The cell e.s.d.'s are taken into account individually in the estimation of e.s.d.'s in distances, angles and torsion angles; correlations between e.s.d.'s in cell parameters are only used when they are defined by crystal symmetry. An approximate (isotropic) treatment of cell e.s.d.'s is used for estimating e.s.d.'s involving l.s. planes.
Refinement. Refinement of *F*^2^ against ALL reflections. The weighted *R*-factor *wR* and goodness of fit *S* are based on *F*^2^, conventional *R*-factors *R* are based on *F*, with *F* set to zero for negative *F*^2^. The threshold expression of *F*^2^ > σ(*F*^2^) is used only for calculating *R*-factors(gt) *etc*. and is not relevant to the choice of reflections for refinement. *R*-factors based on *F*^2^ are statistically about twice as large as those based on *F*, and *R*- factors based on ALL data will be even larger.

### Fractional atomic coordinates and isotropic or equivalent isotropic displacement parameters (Å^2^)

**Table:**

	*x*	*y*	*z*	*U*_iso_*/*U*_eq_	
S1	0.23509 (5)	0.60717 (4)	0.59551 (4)	0.02881 (14)	
Cl1	−0.24623 (5)	−0.05881 (5)	0.30214 (4)	0.03438 (14)	
O1	−0.01441 (13)	0.25159 (11)	0.84778 (9)	0.0258 (2)	
N1	0.03004 (15)	0.36655 (13)	0.66166 (11)	0.0201 (3)	
H1	−0.014 (2)	0.3701 (19)	0.5889 (18)	0.025 (4)*	
N2	0.24161 (15)	0.47809 (13)	0.81460 (11)	0.0215 (3)	
H2	0.192 (2)	0.412 (2)	0.8612 (19)	0.034 (5)*	
C1	−0.04406 (17)	0.25179 (15)	0.73206 (13)	0.0192 (3)	
C2	0.16893 (17)	0.48027 (14)	0.69839 (13)	0.0190 (3)	
C11	−0.16148 (16)	0.12650 (14)	0.65810 (13)	0.0190 (3)	
C12	−0.15027 (17)	0.09819 (15)	0.52694 (13)	0.0203 (3)	
H12	−0.0703	0.1612	0.4809	0.024*	
C13	−0.25894 (18)	−0.02426 (15)	0.46557 (14)	0.0232 (3)	
C14	−0.37636 (19)	−0.11864 (16)	0.53128 (16)	0.0280 (3)	
H14	−0.4495	−0.2015	0.4877	0.034*	
C15	−0.38527 (18)	−0.09009 (16)	0.66142 (16)	0.0280 (3)	
H15	−0.4650	−0.1538	0.7071	0.034*	
C16	−0.27787 (18)	0.03152 (16)	0.72518 (14)	0.0238 (3)	
H16	−0.2837	0.0499	0.8143	0.029*	
C21	0.39206 (17)	0.58278 (15)	0.86417 (13)	0.0194 (3)	
C22	0.55031 (18)	0.56849 (15)	0.82648 (13)	0.0213 (3)	
C23	0.69413 (18)	0.67134 (16)	0.88045 (14)	0.0240 (3)	
C24	0.67324 (19)	0.78059 (16)	0.96980 (14)	0.0255 (3)	
H24	0.7701	0.8486	1.0066	0.031*	
C25	0.5146 (2)	0.79247 (17)	1.00630 (14)	0.0280 (3)	
H25	0.5034	0.8679	1.0671	0.034*	
C26	0.37181 (18)	0.69272 (16)	0.95296 (14)	0.0243 (3)	
H26	0.2624	0.6996	0.9768	0.029*	
C27	0.5701 (2)	0.44874 (18)	0.73006 (16)	0.0325 (4)	
H27A	0.4650	0.3736	0.7199	0.049*	
H27B	0.6625	0.4049	0.7598	0.049*	
H27C	0.5956	0.4901	0.6480	0.049*	
C28	0.8687 (2)	0.6643 (2)	0.84115 (18)	0.0412 (4)	
H28A	0.9513	0.7479	0.8823	0.062*	
H28B	0.8720	0.6671	0.7484	0.062*	
H28C	0.8961	0.5732	0.8672	0.062*	

### Atomic displacement parameters (Å^2^)

**Table:**

	*U*^11^	*U*^22^	*U*^33^	*U*^12^	*U*^13^	*U*^23^
S1	0.0257 (2)	0.0279 (2)	0.0257 (2)	−0.00979 (15)	−0.00909 (14)	0.01144 (15)
Cl1	0.0333 (2)	0.0398 (2)	0.0276 (2)	0.00868 (17)	−0.00649 (16)	−0.01303 (16)
O1	0.0274 (5)	0.0271 (5)	0.0184 (5)	−0.0040 (4)	−0.0013 (4)	0.0031 (4)
N1	0.0201 (6)	0.0193 (6)	0.0172 (6)	−0.0028 (4)	−0.0054 (5)	0.0021 (4)
N2	0.0202 (6)	0.0212 (6)	0.0188 (6)	−0.0040 (5)	−0.0040 (5)	0.0030 (5)
C1	0.0175 (6)	0.0190 (6)	0.0199 (7)	0.0015 (5)	0.0001 (5)	0.0016 (5)
C2	0.0171 (6)	0.0180 (6)	0.0201 (7)	0.0008 (5)	−0.0019 (5)	0.0007 (5)
C11	0.0159 (6)	0.0166 (6)	0.0234 (7)	0.0020 (5)	−0.0026 (5)	0.0019 (5)
C12	0.0179 (6)	0.0182 (6)	0.0238 (7)	0.0026 (5)	−0.0021 (5)	0.0008 (5)
C13	0.0227 (7)	0.0216 (7)	0.0253 (7)	0.0078 (5)	−0.0061 (6)	−0.0046 (6)
C14	0.0222 (7)	0.0184 (7)	0.0395 (9)	−0.0007 (5)	−0.0090 (6)	−0.0025 (6)
C15	0.0215 (7)	0.0211 (7)	0.0384 (9)	−0.0023 (6)	−0.0023 (6)	0.0078 (6)
C16	0.0215 (7)	0.0226 (7)	0.0255 (7)	0.0009 (5)	−0.0006 (6)	0.0046 (6)
C21	0.0206 (7)	0.0189 (6)	0.0163 (6)	0.0002 (5)	−0.0050 (5)	0.0023 (5)
C22	0.0236 (7)	0.0211 (7)	0.0184 (7)	0.0041 (5)	−0.0029 (5)	0.0011 (5)
C23	0.0204 (7)	0.0285 (7)	0.0220 (7)	0.0032 (6)	−0.0033 (5)	0.0038 (6)
C24	0.0235 (7)	0.0249 (7)	0.0240 (7)	−0.0014 (6)	−0.0084 (6)	0.0014 (6)
C25	0.0321 (8)	0.0252 (7)	0.0245 (7)	0.0056 (6)	−0.0055 (6)	−0.0073 (6)
C26	0.0206 (7)	0.0286 (7)	0.0229 (7)	0.0053 (6)	−0.0018 (5)	−0.0021 (6)
C27	0.0316 (8)	0.0343 (8)	0.0311 (8)	0.0092 (7)	0.0010 (6)	−0.0083 (7)
C28	0.0225 (8)	0.0568 (11)	0.0410 (10)	0.0047 (7)	−0.0002 (7)	−0.0057 (8)

### Geometric parameters (Å, °)

**Table:**

S1—C2	1.6751 (14)	C16—H16	0.9500
Cl1—C13	1.7450 (15)	C21—C26	1.394 (2)
O1—C1	1.2315 (17)	C21—C22	1.395 (2)
N1—C1	1.3871 (18)	C22—C23	1.417 (2)
N1—C2	1.3974 (17)	C22—C27	1.511 (2)
N1—H1	0.836 (19)	C23—C24	1.395 (2)
N2—C2	1.3377 (18)	C23—C28	1.511 (2)
N2—C21	1.4472 (17)	C24—C25	1.388 (2)
N2—H2	0.86 (2)	C24—H24	0.9500
C1—C11	1.4966 (18)	C25—C26	1.395 (2)
C11—C16	1.3986 (19)	C25—H25	0.9500
C11—C12	1.402 (2)	C26—H26	0.9500
C12—C13	1.3930 (19)	C27—H27A	0.9800
C12—H12	0.9500	C27—H27B	0.9800
C13—C14	1.392 (2)	C27—H27C	0.9800
C14—C15	1.390 (2)	C28—H28A	0.9800
C14—H14	0.9500	C28—H28B	0.9800
C15—C16	1.393 (2)	C28—H28C	0.9800
C15—H15	0.9500		
			
C1—N1—C2	127.82 (12)	C26—C21—C22	122.41 (13)
C1—N1—H1	117.0 (12)	C26—C21—N2	117.43 (13)
C2—N1—H1	115.2 (12)	C22—C21—N2	120.12 (12)
C2—N2—C21	123.54 (12)	C21—C22—C23	117.98 (13)
C2—N2—H2	116.1 (13)	C21—C22—C27	121.75 (13)
C21—N2—H2	120.3 (13)	C23—C22—C27	120.27 (13)
O1—C1—N1	122.49 (12)	C24—C23—C22	119.38 (13)
O1—C1—C11	121.81 (12)	C24—C23—C28	120.03 (14)
N1—C1—C11	115.70 (12)	C22—C23—C28	120.59 (14)
N2—C2—N1	116.65 (12)	C25—C24—C23	121.69 (13)
N2—C2—S1	124.50 (10)	C25—C24—H24	119.2
N1—C2—S1	118.84 (10)	C23—C24—H24	119.2
C16—C11—C12	120.14 (12)	C24—C25—C26	119.50 (14)
C16—C11—C1	117.91 (12)	C24—C25—H25	120.2
C12—C11—C1	121.84 (12)	C26—C25—H25	120.2
C13—C12—C11	118.60 (13)	C21—C26—C25	119.04 (13)
C13—C12—H12	120.7	C21—C26—H26	120.5
C11—C12—H12	120.7	C25—C26—H26	120.5
C14—C13—C12	121.68 (14)	C22—C27—H27A	109.5
C14—C13—Cl1	119.67 (11)	C22—C27—H27B	109.5
C12—C13—Cl1	118.64 (12)	H27A—C27—H27B	109.5
C15—C14—C13	119.17 (13)	C22—C27—H27C	109.5
C15—C14—H14	120.4	H27A—C27—H27C	109.5
C13—C14—H14	120.4	H27B—C27—H27C	109.5
C14—C15—C16	120.31 (14)	C23—C28—H28A	109.5
C14—C15—H15	119.8	C23—C28—H28B	109.5
C16—C15—H15	119.8	H28A—C28—H28B	109.5
C15—C16—C11	120.08 (14)	C23—C28—H28C	109.5
C15—C16—H16	120.0	H28A—C28—H28C	109.5
C11—C16—H16	120.0	H28B—C28—H28C	109.5
			
C2—N1—C1—O1	14.4 (2)	C12—C11—C16—C15	−1.2 (2)
C2—N1—C1—C11	−164.92 (13)	C1—C11—C16—C15	−177.41 (13)
C21—N2—C2—N1	176.32 (12)	C2—N2—C21—C26	103.83 (16)
C21—N2—C2—S1	−2.7 (2)	C2—N2—C21—C22	−78.45 (18)
C1—N1—C2—N2	1.0 (2)	C26—C21—C22—C23	−0.6 (2)
C1—N1—C2—S1	−179.91 (11)	N2—C21—C22—C23	−178.24 (12)
O1—C1—C11—C16	20.3 (2)	C26—C21—C22—C27	−179.96 (14)
N1—C1—C11—C16	−160.43 (12)	N2—C21—C22—C27	2.4 (2)
O1—C1—C11—C12	−155.86 (13)	C21—C22—C23—C24	1.0 (2)
N1—C1—C11—C12	23.43 (19)	C27—C22—C23—C24	−179.67 (14)
C16—C11—C12—C13	1.1 (2)	C21—C22—C23—C28	−178.42 (14)
C1—C11—C12—C13	177.14 (12)	C27—C22—C23—C28	0.9 (2)
C11—C12—C13—C14	−0.5 (2)	C22—C23—C24—C25	−0.8 (2)
C11—C12—C13—Cl1	179.51 (10)	C28—C23—C24—C25	178.63 (15)
C12—C13—C14—C15	−0.1 (2)	C23—C24—C25—C26	0.2 (2)
Cl1—C13—C14—C15	179.96 (11)	C22—C21—C26—C25	0.0 (2)
C13—C14—C15—C16	0.0 (2)	N2—C21—C26—C25	177.71 (13)
C14—C15—C16—C11	0.7 (2)	C24—C25—C26—C21	0.2 (2)

### Hydrogen-bond geometry (Å, °)

**Table:**

*D*—H···*A*	*D*—H	H···*A*	*D*···*A*	*D*—H···*A*
N2—H2···O1	0.86 (2)	1.99 (2)	2.6840 (16)	137.7 (17)
N1—H1···S1^i^	0.836 (19)	2.636 (19)	3.4376 (13)	161.2 (15)

Symmetry codes: (i) −*x*, −*y*+1, −*z*+1.
